# Canine-Assisted Therapy Improves Well-Being in Nurses

**DOI:** 10.3390/ijerph16193670

**Published:** 2019-09-30

**Authors:** Kristýna Machová, Michaela Součková, Radka Procházková, Zdislava Vaníčková, Kamal Mezian

**Affiliations:** 1 Department of Ethology and Companion Animal Science, Faculty of Agrobiology, Food and Natural Resources Czech University of Life Sciences, 16500 Prague, Czech Republic; souckovamichaela@af.czu.cz; 2 Department of Statistics, Faculty of Economics and Management, Czech University of Life Sciences, 16500 Prague, Czech Republic; prochazkova@pef.czu.cz; 3 Institute of Medical Biochemistry and Laboratory Diagnostics, Charles University, First Faculty of Medicine, 12808 Prague, Czech Republic; zdislava.vanickova@lf1.cuni.cz; 4 Department of Rehabilitation Medicine, Charles University, First Faculty of Medicine, 12108 Prague, Czech Republic; kamal.mezian@gmail.com

**Keywords:** Healthcare providers, animal-assisted therapy, dog-assisted therapy, stress, cortisol

## Abstract

As nursing is one of the most stressful occupations worldwide, its management warrants more attention to identify possible ways to cope with its pressures. This study aims to evaluate whether animal-assisted therapy (AAT) with the presence of a dog affects the stress level of nurses. As a stress biomarker, we used salivary cortisol level testing. Twenty female nurses (mean age: 30) in physical medicine (PMR) (*n* = 11) and the department of internal medicine and long-term care (IM < C) (*n* = 9). On each of the three observed days, saliva was collected at 10 a.m. and then again after 50 min. The first sampling was performed during a normal working process without a break (Condition A), the second was carried out during a normal working process with a break of choice (Condition B), and the third sampling was performed during a normal working process with a break with AAT (Condition C). All participants were enrolled in all three interventional conditions in a randomized order. The results demonstrated the effect of a reduction of cortisol levels in Condition C, where AAT was included (*p* = 0.02) only in nurses recruited from the IM < C department. By way of explanation, nurses from the PMR department already showed low cortisol levels at baseline. We propose including AAT with a dog in healthcare facilities where nurses are at a high risk of stress.

## 1. Introduction

In recent decades, much attention has been paid to the impact of stress on human health and possible methods of stress control [1]. Short-term, mild, and stimulating stress may have a positive effect on humans. In this case, we are talking about eustress, which has sometimes been neglected in the literature [2]. Though its conception is incomplete [3], one of its described characteristics is the initiation of the organism’s adaptive capacities to stress. On the other hand, distress is a type of stress to which the individual finds himself or herself inadequately able to respond, to which response is unsustainable in the long term, and which results in impaired adaptation [4].

The factor determining whether a stressor will cause eustress or distress is an individual’s perception and interpretation of a situation. The perception of stress is also determined by its duration, its source, manageability, and suitability [5]. Nelson and Simmons [3] assumed that eustress and distress can concurrently occur. Nevertheless, there is only one physiological response to stress in an organism, although the ratio and the number of structural elements involved in the reaction may vary [6]. From here, we can discuss different reactions to stressors.

The term “stress” has several different meanings [7]. Psychogenic stress occurs when an individual is exposed to psychological or social challenges which lead to the disruption of his/her psychological well-being [8]. Healthcare providers are mainly exposed to this type of stress but often face stress caused by the work structure itself. Twelve-hour work shifts, alternating day and night shifts, occasionally physically strenuous work, and engaging with ill and dying patients as well as their families are all stressful factors healthcare personnel have to face [9,10]. If the activation of a stressful reaction is increased or prolonged, inappropriate immunosuppression can occur [11], which may lead to a higher risk of infection and neoplasia, as well to lower reactions to vaccinations [12]. Healthcare and caring staff may then be considered an endangered group where the possible effect of animal-assisted therapy (AAT) can be investigated because of the psychological demands of their occupation and a higher exposure to infectious diseases.

At present, the inclusion of AAT in hospitals and healthcare facilities is no longer an exception [13]. The possible effects of AAT include the ones on human physiology like the reduction of heart rate [14] and cortisol level [15], improvements in blood pressure control [16,17], and a decrease of Immunoglobulin A level. In addition, an increase in oxytocin levels may result [18]. AAT also affects the psychosocial aspects of human life. It can be included in the treatment of depression, anxiety, and other psychosocial diseases, as it helps one to experience joy, a sense of relief, and relaxation [19].

It is precisely because of the availability of this potential possibility of stress reduction that the inclusion of AAT may be appropriate not only among patients but also among the healthcare and caring staff, thus helping to improve their mental and physical well-being. The aim of this study was to evaluate whether AAT would reduce cortisol levels (a biological marker of stress) in nursing staff working in a hospital setting.

## 2. Methods

### 2.1. Participants

The cohort comprised 22 women working at the Central Military Hospital in the Czech Republic (the mean age was 30 years). These nurses were recruited from two different departments, the first of which was the department of rehabilitation and physical medicine (*n* = 13; 2 of them excluded), and the second was the department of internal medicine and long-term care (*n* = 9). Those considered suitable were women working as nurses and assistant medical workers who had been working in the given department for more than one month and had been in this profession for more than one year. Women were considered appropriate with the intention to reach the sample homogeneity in light of previous studies that have shown different cortisol responses to psychological stress in men and women. Additionally, the vast majority of healthcare providers (HCPs) in our settings were women. Those excluded were women who were pregnant, in menopause, or presented with acute illness at the time (pregnancy, *n* = 1; herpes zoster infection, *n* = 1). Women who had a positive attitude to dogs and who agreed to participate in this study were deemed suitable candidates. Prior to participation by said candidates, the authors obtained their informed consent [20]. The project was approved by the Ethics Committee of the Central Military Hospital in Prague (Military University Hospital)—reference number 108/12-89/2018. Dog participation on AAT was approved by the Ethics Committee of the Czech University of Life Sciences in Prague – reference number 05/17. The study and its methodological procedure adhered with the requirements of European Union and Czech legislation (Act no. 246/1992 coll. on animal protection as amended by Act no. 162/1993 coll.).

### 2.2. Measures

The vast majority of existing stress measurement tools are based primarily on the retrospective assessment of perceived stress. The disadvantage of this measurement is that stress assessment can be influenced by memory, verbal abilities, interoceptive awareness, or personality traits [21]. For maximum objectively, cortisol levels from saliva were used to determine the AAT effect. This method of measurement is the most widespread hormonal measurement of psychogenic stress commonly used in humans and dogs [22]. The concentration of cortisol in plasma, saliva or urine markedly increases 15–30 min after the onset of stress [23]. The physiological range of salivary cortisol in adult women is 0.3–12ng/mL at the selected time of day.

The medical staff did not consume food or liquids (except water) for at least 30 min before collection and during the observed period. Smoking and the brushing of teeth before the collection were also prohibited. The work shift started for all nurses at 7 o´clock.

Saliva samples were collected according to the manufacturer’s instructions ((DRG Salivary Cortisol ELISA (SLV-2930) (DRG International, Inc., USA)), as were sample processing, storage, the selection of the analytical method, and the determination of appropriate reference intervals [24].

Using a special saliva sampling straw, a sterile absorbent swab was placed in the participant’s mouth for 30–120s [25] and chewed gently to facilitate absorption of saliva. The swab was then placed into a sterile centrifuge tube and securely sealed [24,26]. After collection, the saliva samples were immediately sent to the on-site laboratory. There, the samples were centrifuged at 3000 rpm for 15 min, and the supernatant collected was stored at –20 °C for further analysis. Within 30 days, the samples were transported in a dry ice box to the Institute of Medical Biochemistry and Laboratory Diagnostics for the analysis of the salivary cortisol concentration using the ELISA. Before the assay, the samples were thawed at room temperature (21–26 °C).

### 2.3. General Procedures

In this study, we used a cross-over design. All participants were subsequently enrolled in all three interventional conditions in a randomized order. For the randomization, we used a sealed/opaque envelope containing three notes, each of them indicating one of the three intervention situations (A, B or C). Before the procedure, each of the participants drew a random note from her envelope to be accordingly allocated to the intervention situation. On each of the three observed days, saliva was collected at 10 a.m. and then again after 50 min. The saliva samples were collected on a working day with the condition of overnight sleep before the day of collection. The first sampling was performed during a normal working process without a break (Condition A), the second was carried out during a normal working process with a break of choice (Condition B — for example, reading, talking to friends, or checking their phones), and the third sampling was performed during a normal working process with a break during which AAT in the presence of a dog took place (Condition C). The interaction with the dog was uncontrolled and lasted 20 min, during which only the observed individual, the dog, and its handler were present in a quiet room reserved for this study. During this interaction, the content of the session was decided by the participants who had the opportunity to pet the dog, feed her, or play with her. During the reporting period, the participants were on a mattress where they had the opportunity to sit, lie, or just be next to the dog.

### 2.4. Therapy Dog

The therapy dog was a female Border Collie, Mia, who had been working regularly in the hospital for 3 years. This dog had passed exams to perform animal-assisted therapy and has been regularly vaccinated and dewormed. The therapy dog had access to water ad libitum, as well as sufficient room and break times in which to rest or play. She also showed behaviors that suggested that she enjoyed the therapeutic encounters.

### 2.5. Data Analysis

The measured data were analyzed according to the conditions (A, B, or C) depending on how the medical staff (respondents) tried to reduce their stress levels: Condition A—without a work break; Condition B—with a work break of their choice; and Condition C—with animal-assisted therapy. The data were also compared between the two hospital wards.

At baseline, an exploratory data analysis was conducted to verify the assumptions for subsequent processing (such as sampling independence, homogeneity and distribution normality). The premise was the normal Gaussian distribution, which was assessed by the Shapiro–Wilk test and further evaluated on histograms and normalized probability diagrams.

With respect to the number of respondents (n = 20), the analyzed data did not show a normal Gaussian distribution in most cases. Some observations appeared to be remote or extreme within the analyzed sets; therefore, non-parametric procedures were primarily used to test and generalize the significance of differences in measured cortisol values. Wilcoxon’s paired test for dependent samples was used to test differences in cortisol levels within each condition (differences in values measured at 10:00 a.m. and 10:50 a.m.). The Kruskal–Wallis test was used to assess the differences in the results of Conditions A, B, and C.

Parametric procedures were used to compare measurement results in Conditions A, B, and C within each of the two analyzed hospital workplaces. Since the data showed a normal Gaussian distribution and the Levene test showed variance homogeneity, a single-factor ANOVA was used.

Parametric tests could also be used to examine the conclusiveness of the differences between the two departments. Evidence of difference in mean changes in cortisol levels was tested using a two-sample t-test, and the difference in the variability of cortisol changes was evaluated by an F-test. Statistical significance was set at *p* < 0.05.

Box charts, graphs of means, and reliability intervals were used to present the results of statistical analyses.

## 3. Results

### 3.1. Overall Results of Cortisol Change Evaluation

**Figure 1:**
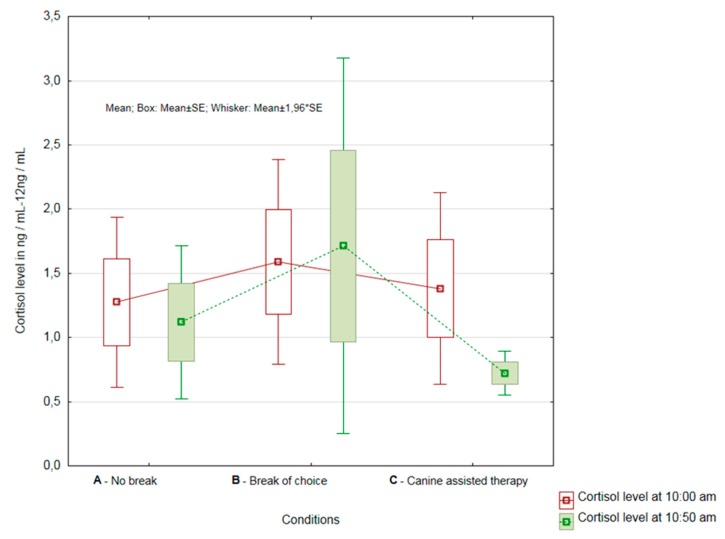
Overall results of cortisol change evaluation in Conditions A, B and C.

The results showed that there was a significant difference in the complete group of nurses (*n* = 19), demonstrating the effect on the reduction of cortisol levels in Condition C, i.e., the condition where AAT was included (*p* = 0.02) (see Figure 1).

**Figure 2:**
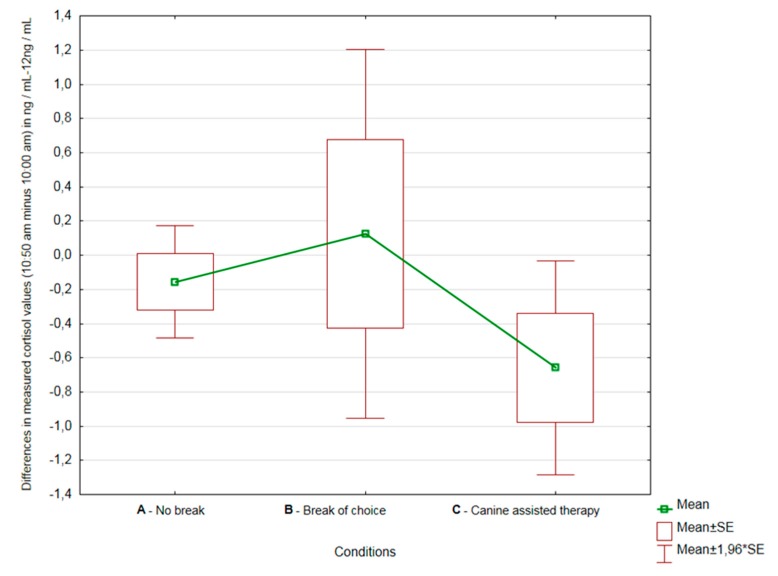
Differences in measured cortisol values (10:50 a.m. minus 10:00 a.m.) in Conditions A, B and C.

Not only was the cortisol level statistically lower after the second sampling, the measured values showed less variability as well. It is also apparent from Figure 2 that, on average, the greatest decreases in cortisol levels were observed in Condition C.

### 3.2. Comparison of Results by the Department of Healthcare Providers

#### 3.2.1. Healthcare Providers from the Department of Rehabilitation

**Figure 3:**
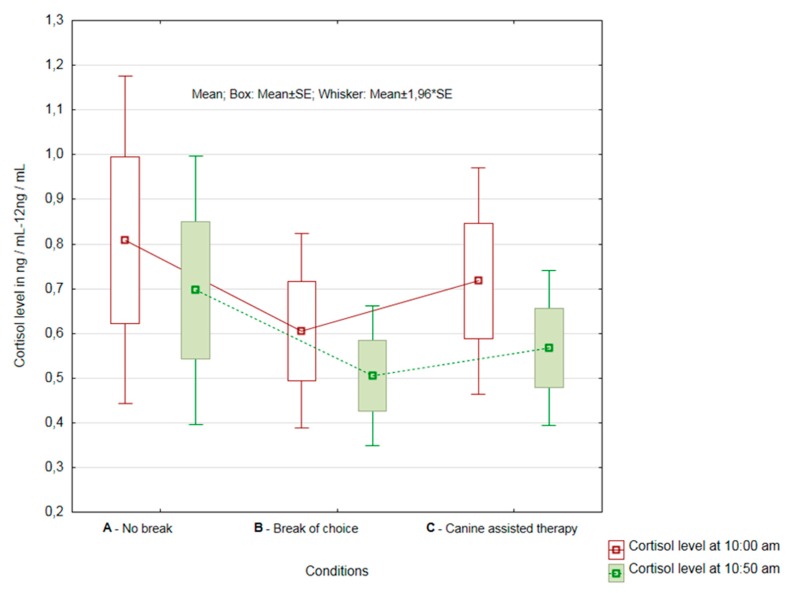
Results of cortisol change evaluation in Conditions A, B and C at the department of physical and rehabilitation medicine.

Since the nurses were from two departments, we decided to compare the results of nurses within the individual departments as well. The results showed that there was no statistically significant difference between the first collection at 10:00 and the second collection at 10:50 in the case of HCPs in the rehabilitation department (department of physical and rehabilitation medicin—PRM) (n = 11) (Condition A—*p* = 0.09; Condition B—*p* = 0.34; and Condition C—*p* = 0.08). Significant reductions in cortisol levels after AAT were observed in individual nurses. However, the magnitude of the recalculated significance levels (*p*) showed that Condition C was the closest to the rejection of the null hypothesis and demonstrated a statistically significant difference (see Figure 3).

When evaluating the significance of the magnitude of changes in cortisol levels between all three conditions, there was no difference in the mean magnitude of changes (*p* = 0.83) or the variability of cortisol level changes (*p* = 0.13).

#### 3.2.2. Healthcare Providers from the Department of Internal and Long-Term Care

**Figure 4:**
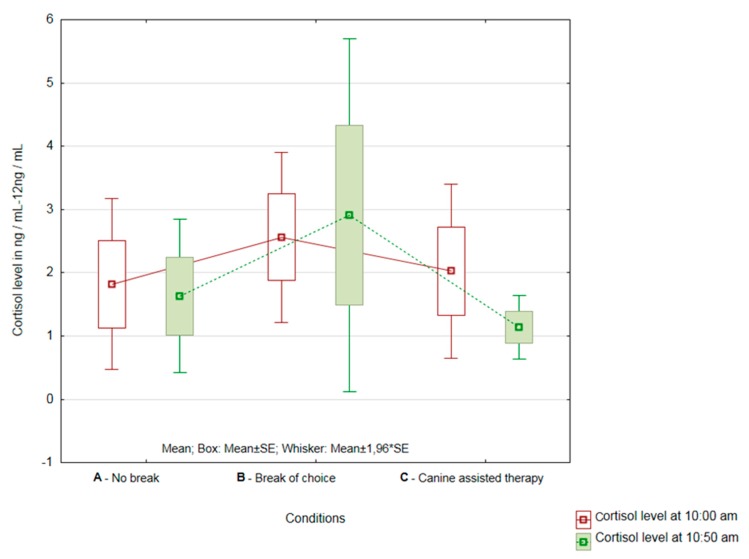
Results of cortisol change evaluation in Conditions A, B and C at the department of internal medicine and long-term care.

In HCPs from the department of internal medicine and long-term care (*n* = 9), the differences between the individual conditions tested were greater than in the HCP group from the department of rehabilitation. In this group, there was a significant difference in the AAT inclusion (Condition C—*p* = 0.0499). There was no statistically significant reduction in cortisol levels in Condition A (no break) and Condition B (with a break of choice) (A—*p* = 0.37; B—*p* = 0.96) (see Figure 4). However, even in this group of HCPs, a significant decrease in cortisol levels was observed after the completion of AAT in individual nurses.

When evaluating the significance of the magnitude of changes in cortisol levels between all three conditions, there was no difference in the mean magnitude of changes (*p* = 0.56) or the variability of cortisol levels changes (*p* = 0.13).

#### 3.2.3. Assessment of the Significance of Differences in Results of both Groups of Respondent-Departments

**Figure 5:**
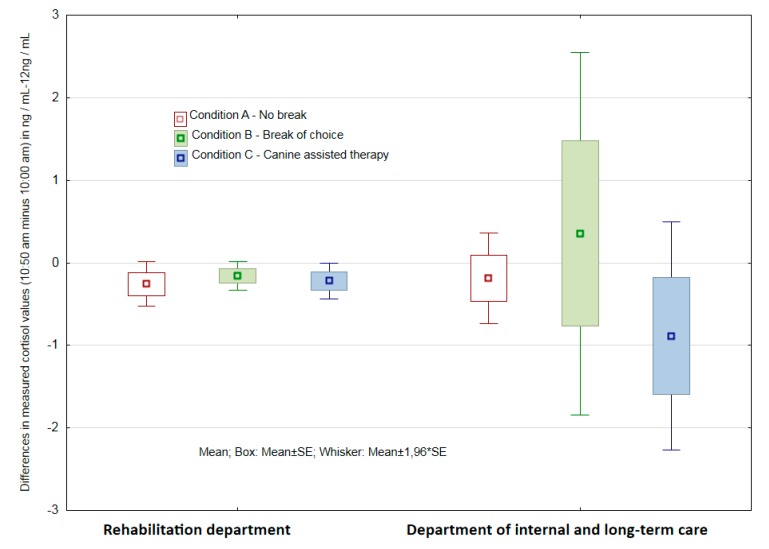
Differences in measured cortisol values (10:50 a.m. minus 10:00 a.m.) in Conditions A, B and C at the department of physical and rehabilitation medicine and at the department of internal medicine and long-term care.

For Condition A (no break), a two-sample t-test showed no statistically significant difference in the mean changes in cortisol levels (*p* = 0.87), and the F-test showed no difference in the variation of changes (*p* = 0.55). There was no statistically significant difference in mean changes in cortisol levels (*p* = 0.69) for Condition B (with a break of choice), but in this case, a statistically significant variance in the variability in cortisol level changes was shown by the F-test (*p* < 0.001). The PMR department HCPs with 95% reliability showed statistically a significantly lower variability of changes. There was no statistically significant difference in the mean changes in cortisol levels (*p* = 0.292) for Condition C (AAT). However, from the magnitude of the recalculated levels of significance (*p*), it can be concluded that Condition C was the closest to the rejection of the null hypothesis and demonstrated a statistically significant difference. This condition also exhibited a statistically significantly lower variation of change with 95% reliability (*p* < 0.001) (see Figure 5).

## 4. Discussion

In the present study, as in previous work (Lass-Hennemann [15]; Rodriquz et al. [27]), salivary cortisol was reduced in health care providers after undergoing animal-assisted therapy. The altruistic nature of HCPs may often be the cause of excessive work. Very often, medical staff, especially nurses, have a clear professional and life goal, which is to care for others [28]. Hart [29] stated that nurses see the greatest accomplishment of their work in helping patients and their families (62%). Healthcare professionals must usually respond quickly and under pressure, have a great deal of responsibility, and often have to improvise [9,30,31].

Stress regulation and burnout prevention include the need to create boundaries between professional and private life [32,33]. Coping with relaxation techniques and regular mental hygiene is often essential [34].

Regular visits to the AAT program may provide individually selected HCPs contact with a pet, an efficiently spent break, a positive experience in the working environment, and space to disclose concerns to someone in a pleasant environment [35]. The presence of an animal could also be a helpful incentive to participate in collective supervisions [36].

An interesting finding of this study was the different effectiveness of AAT in HCPs from different wards. The cortisol levels of nurses working in the rehabilitation and physical medicine department did not decrease after AAT, while a decrease was observed in HCPs working in the internal medicine ward, which included the long-term care department. A likely explanation for this difference was the very low initial cortisol value of HCPs from the PRM department, which was around 0.1 ng/mL. In light of the aforementioned low-cortisol level of HCPs in the PRM department, we speculate that the workload of HCPs in the PRM department was less stressful than it was in the internal medicine and long-term care department.

In contrast, the initial cortisol values in nurses from the internal ward were, on average, around 3.5 ng/mL and therefore had space to lower this level. Thus, it appears that AAT may have different effects in various departments. According to Bukhari et al. [37], the largest proportion of medical staff with depression symptoms appear in the Intensive care unit (ICU), coronary units, emergency rooms, and operating rooms, and it seems that workers from these departments should be able to use AAT for its beneficial effects.

HCPs are also generally more at risk of depression. Letvak, Ruhm, and McCoy [38] reported up to twice the incidence of depression (18% vs. 9.4%) compared to the general population, and, furthermore, Welsch [39] reported signs of mild-to-moderate depression in as much as 35% of medical staff. The possible effect of AAT on reducing depressive symptoms has been described several times [40]. In these sessions, it is therefore possible to expect not only a decrease in cortisol levels but also a potential decrease in depression.

Surprisingly, a decrease in cortisol levels was not observed in HCPs who had a break of their choice in neither of the two departments. In the study by Veiga et al. [21], the reduction of cortisol was observed during relaxation interventions, and this study showed that a psychomotor relaxation program could be an efficient tool for managing the work-related stress of healthcare professionals. However, the HCPs who participated in this study either did not know how to use these techniques or did not include them in their preferred form of a break. It seems likely that in AAT, HCPs were more focused on the moment and therefore showed a greater reduction in cortisol levels in the third observed condition.

Medical staff often encounter shift rotation systems that significantly affect their circadian rhythms and that are known to be deleterious to mental and physical well-being. Previous cross-sectional studies have demonstrated a contrast between the results of cortisol levels and working day and night shifts [41]. Li et al. [41] reported that shiftwork greatly altered the circadian cortisol model and is predictive of increased cortisol levels. In the study by Copertaro et al. [42], however, none of the variables studied differed significantly between night shift or day shift workers, and no effect of shifts on the immune system and cortisol levels was observed. Additionally, because of the uncertainty about the effect of shift rotation system on cortisol levels, further investigation, as well as a further observation of the effect of AAT in individual departments, is needed to maximize the efficiency of AAT and the support of HCPs.

Our results show that AAT could be part of preventive programs for this group of employees, which may be exposed to significant stress daily [43], and AAT could help to create a strategy to reduce it.

## 5. Limits

The limit of this study is that the people present were HCPs who were willing to interact with the dog, and, furthermore, the participants were only female. This was because the HCPs were in most cases women, and, according to literature, it has been observed that more positive reactions to AAT occur in women than in men. These results may not generalize to other populations. Investigating the impact of AAT on male healthcare providers could be the next step, as could more extensive observation with more healthcare providers.

Another restriction could be the effect of the social skills of the dog handler; even though the conditions were the same for all participants, it is still possible that the personal qualities of the dog handler had an impact on the stress level of HCPs.

Designation Animal-assisted therapy was used in this case, although this was only a single visit to HCPs. In the case of implementation, there would be more regular meetings led by a professional therapist with a clearly stated objective of preventing or supporting the management of stress at work.

## 6. Conclusions

In this interventional study, assessing the impact of AAT on HCPs, we found a statistically significant reduction of the salivary cortisol level in HCPs working in the internal medicine and long-term care department. AAT could be included as one of the strategies to help prevent or reduce the stress of HCPs.
